# A novel transdermal ketoprofen formulation for analgesia in cattle

**DOI:** 10.1111/jvp.13093

**Published:** 2022-09-04

**Authors:** Paul C. Mills, Jane G. Owens, James B. Reinbold, Michael McGowan, Claudia Ellenbergner, Solomon Woldeyohannes, Nana Satake

**Affiliations:** ^1^ School of Veterinary Science The University of Queensland Gatton Australia; ^2^ JGO Consulting, LLC Indianapolis Indiana USA; ^3^ Elanco Animal Health Greenfield Indiana USA; ^4^ Elanco Animal Health Australia Australia

**Keywords:** analgesia, ketoprofen, pharmacokinetics, tracsdermal

## Abstract

Ketoprofen is registered in many countries for injectable administration in cattle. Because it is soluble in a wide range of excipients, development of a novel transdermal (TD) ketoprofen formulation was pursued to provide a convenient and pain‐free route of administration in cattle. One hundred and six excipient combinations were screened using in vitro techniques (Franz diffusion cells), with a 20%_(w/v)_ ketoprofen formulation dissolved in a combination of 45%:45%_(v/v)_ ethanol and isopropyl myristate (IPM) and 10%_(v/v)_ eucalyptus oil achieving maximal penetration of ketoprofen through bovine skin. A bioavailability study was then conducted using a randomized cross‐over design (*n* = 12), including IV, IM (both 3 mg/kg) and TD (10 mg/kg) ketoprofen formulations administered with a one‐week washout period between administrations. The IV and IM formulation pharmacokinetic results were as expected. The *C*_MAX_, *T*_max and_ AUC_0‐Last_ were significantly higher (arithmetic mean ± SD) after TD administration (20.0 ± 6.5 μg/ml, 115 ± 17 min and 3940 ± 1324 μg*min/ml, respectively), compared to IM (11.0 ± 4.0 μg/ml, 74 ± 43 min and 2376 ± 738 μg*min/ml, respectively), although there were no significant differences for T_½β_. However, dose corrected values *C*_MAX_ and AUC_inf_ were significantly higher for IM compared to TD. The arithmetic mean bioavailability (F) of the transdermal formulation was 50%. The plasma concentration of the TD formulation at a dose of 10 mg/kg was similar to the IM formulation at 3 mg/kg by 30 min post‐dosing with an arithmetic mean ± SD of 7.97 ± 4.38 vs. 8.02 ± 3.55 μg/ml, respectively. The TD formulation was generally well tolerated by cattle, although some local irritation along the site of application was noted after 12 h of exposure during the bioavailability study. Results indicate that this novel TD formulation provides a substantial improvement in administration convenience, may improve animal welfare and end‐user safety through needle‐free administration, and achieves similar plasma pharmacokinetics to the IM product when administered at 10 mg/kg.

## 1 INTRODUCTION

Cattle may be subjected to painful procedures in the course of routine husbandry practice, such as dehorning, laparotomies, needle injections, spaying, and surgical and non‐surgical castration of males (Hemsworth & Arnold, 2006; Stafford & Mellor, 2005). Analgesia is often overlooked in farm animals, partly due to prey animals tending to mask pain behaviours and for practical reasons in terms of individual injection of large numbers of cattle and the cost of administration without a return on investment from a sustained live animal performance benefit. Effective analgesia treatment may prevent the development of chronic pain in addition to ameliorating the short‐term nociceptive responses (Viñuela‐Fernández et al., 2007). Additional benefits include minimizing acute production losses (Goonewardene & Hand, 1991; Winks et al., 1977), providing responsible animal care (Viñuela‐Fernández et al., 2007) and serving as an important part of the ‘social licence’ to conduct a production animal business.

In addition to economic considerations, it may be that practical aspects may also limit the use of analgesics in cattle. *En masse* surgical procedures, such as castration and dehorning (Viñuela‐Fernández et al., 2007), may not be managed with an analgesia protocol due to the lack of approved products or processing time required for additional needle injections. Alternatively, TD administration has the potential to address both practical and economic considerations based on a quick, needle‐free ease of administration to cattle by non‐veterinary staff. This leads to a reduction in product costs compared to the rigours and expenses of parenteral formulations (Mills & Cross, 2006). Topically applied non‐steroidal anti‐inflammatory drugs (NSAIDs) are becoming increasingly popular in human medicine, including ketoprofen (Derry et al., 2017; Serinken et al., 2016). Topical NSAIDs also offer a better risk‐to‐benefit ratio than NSAIDs administered by other routes, including oral administration (Rafanan et al., 2018). In Australia, there are extensive cattle production systems with herds of thousands of animals managed on properties with a variety of terrains. Application of analgesics during *en masse* procedures must be easy to apply and have quick to onset of effect. There are no commercially available topical NSAID formulations containing ketoprofen that are registered for use in cattle in Australia, although there are topical formulations containing flunixin meglumine available for use in cattle outside of Australia (Kleinhenz et al., 2016, Kleinhenz, Gorden, et al., 2018, Kleinhenz, Van Engen, et al., 2018). Flunixin meglumine administration has been reported to increase the risk for retained placenta, elevated rectal temperature, decreased milk yield and was associated with an increased incidence of metritis and stillborn calves when compared with controls (Newby et al., 2017).

The current study briefly describes the use of in vitro techniques to screen a wide range of vehicles (excipients) for their potential use for transdermal delivery of ketoprofen. A bioavailability study was then undertaken comparing the leading candidate transdermal formulation to intravenous (IV) and intramuscular (IM) commercially available formulations.

## 2 MATERIALS AND METHODS

### 2.1 Animals

Skin was harvested from Black Angus steers (*n* = 3, age 1.5–2 years old, 5/5 body condition score and weighing 508–570 kg) immediately after slaughter at a local abattoir (JBS Australia Pty Ltd, Dinmore, QLD, Australia). This study was approved by The University of Queensland Animal Ethics Committee (approval certificate SVS/455/14/MLA). The authors confirm that the ethical policies of the journal, as noted on the journal's author guidelines page, have been adhered to and that the appropriate ethical review committee approval has been received. The authors confirm that they have adhered to International Standards for the protection of animals used for scientific purposes.

For the bioavailability study, 6 steers and 6 heifers (5/5 body condition score and weighing 293–352 kg and aged 9–12 months) consisting of Angus, Hereford, Murray Grey, Shorthorn, and Angus‐crossbred beef‐type cattle were used from Elanco's Yarrandoo R and D Centre (Kemps Creek, NSW, Australia) in a randomized, non‐blinded, three‐treatment, 3‐period, 3‐sequence crossover pharmacokinetic study. This cohort of young animals was selected as the target group which would undergo routine husbandry procedures such as dehorning and sterilization. This study was conducted at Elanco Australasia Pty Ltd, approved by the Elanco Animal Ethics Committee and conducted under the APVMA Small‐scale Trials Permit PER7250 and GLP study conditions.

The study was undertaken in June (Winter) with ambient temperatures ranging from 8 to 18°C. Only the cattle receiving the TD formulation were housed individually in covered, outdoor pens on day of treatment administration until the final blood collection was drawn. For all other study days and animals that were not administered the TD formulation, housing occurred in outdoor feedlots where half of the pen is under cover.

### 2.2 In vitro excipient screening

The objective of this component of the investigation was to determine the vehicles that provided the maximum rate and extent of transdermal NSAID absorption.

**FIGURE A1:**
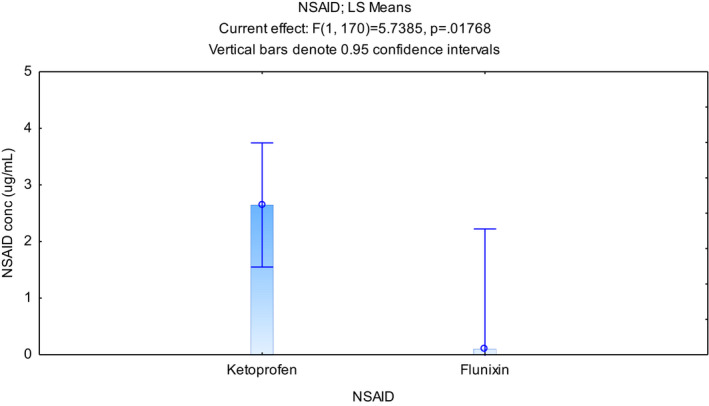
Movement on NSAIDs through cattle skin in vitro

**FIGURE A2:**
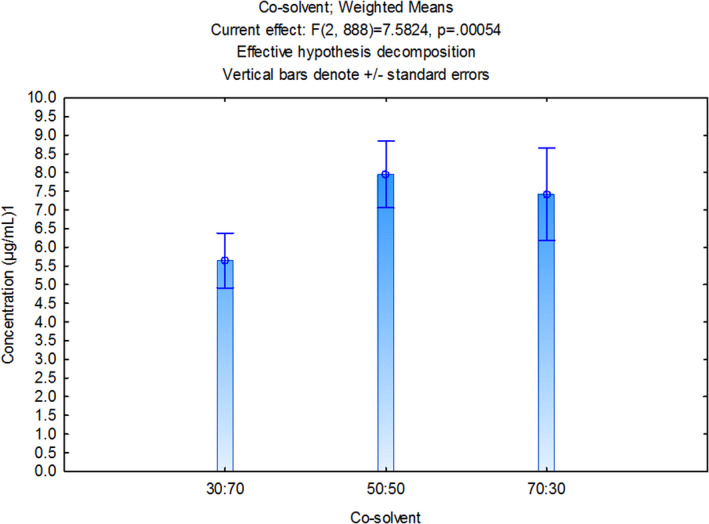
Effects of various concentrations of ethanol on ketoprofen movement through cattle skin

**FIGURE A3:**
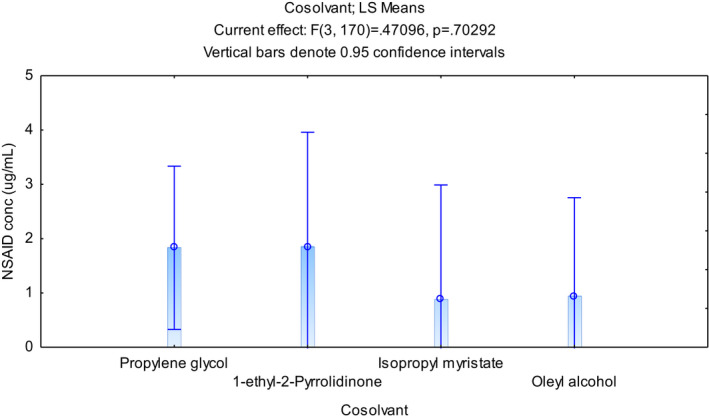
Effects of various solubilising agents on ketoprofen movement through cattle skin

Hair was removed using an electric clipper. Subsequently, the subcutaneous fat and musculature were carefully trimmed before the skin was cut into circular sections (approximately 2 cm diameter), placed in an airtight plastic bag and stored at −20${}^{{}^{\circ}}$C within 5 h of slaughter. The in vitro studies were undertaken within 2 weeks of the skin being frozen. Skin discs (whole skin) were randomly selected, thawed and then placed on a Franz diffusion cell containing phosphate‐buffered saline with 4% bovine serum albumin in the receptor chamber (Mills and Cross, 2007). Ketoprofen sodium salt (purchased from BOVA Australia Pty Ltd, Caringbah, NSW, Australia) was solubilized at 5%, 10%, 20% and 30% _(w/v)_ into 106 excipient preparations consisting of terpenes, alcohols and stabilizers (A Appendix). Three other NSAIDS that were registered for use in cattle in Australia were also investigated. Tolfenamic acid and meloxicam could not be solubilized, so were excluded. Flunixin meglumine could be solubilized but exhibited markedly lower TD penetration as compared to ketoprofen in the irrespective of vehicle (Figures A1, A2, A3 in B Appendix). Consequently, the decision was made to limit our in vitro investigation to vehicles facilitating the TD diffusion of ketoprofen.

A 1.0 ml volume of each formulation was added to the donor chamber of the Franz diffusion cell (replicates of three for each formulation). The recipient chamber was filled with warmed 4% bovine serum albumin in phosphate‐buffered saline, ensuring skin‐to‐solution contact. Then, a 200 μl sample of recipient was removed and replaced at 0, 15, 30 and 60 min, and at 2, 4, 6, 8 and 12 h. These samples were frozen at −20${}^{{}^{\circ}}$C within 15 min of collection and within a further 48 h the samples were defrosted in a batch, the ketoprofen was extracted and the concentration was analysed by liquid chromatography‐mass spectrometry (LC–MS) as described herein.

### 2.3 Analysis

Only the uHPLC‐LCMS/MS analytical method for utilized for ketoprofen analysis is reported here since it was the candidate formulation that was finally selected. Similar methods were also developed for the other NSAIDs. The same method was used both for in vitro and in vivo studies.

Plasma or in vitro samples were mixed with cold acetonitrile (0.2% formic acid) at 1:1 ratio and vortexed for 30 s to precipitate proteins. Proteins were precipitated and removed by two rounds of centrifugation for 15 min at 20,000×*g*, with 30 min at 4°C between each round. The supernatant was then analysed for ketoprofen concentration by tandem uHPLC‐LCMS/MS, with spiked recoveries of ketoprofen of 91.2% to 102.6%, using deuterated ketoprofen as the internal standard. A Nexera™ uHPLC in tandem with LCMS 8030 (Shimadzu Co. Ltd, Tokyo, Japan), with argon as the carrier gas and a Kinetex C18, 1.7 μm pore size column (Phenomenex Inc., Lane Cove, NSW, Australia). The mobile phase A was 0.1% formic acid in water and mobile phase B was 0.1% formic acid in Acetonitrile. Detection m/z 255.10 > 105.20 and confirming ion 255.10 > 194.25. The limit of detection (LOD) was 0.01 μg/ml, and the calibration range was 0.019–5 μg/ml. The QC was determined over a range of concentrations (0.1, 2 and 5 μg/ml) and was between 9% and 11%.

### 2.4 Bioavailability study

Cattle were randomized to three treatment groups (*n* = 4 per group) to receive the IV, IM and transdermal (TD) formulations (treatment) using a randomized three period, three sequence, three treatment crossover design. Each group received a single administration of the following treatments: (1) ketoprofen 3 mg/kg IV (Ilium Ketoprofen 100 mg/ml; Troy Animal Health care, Troy laboratories, Glendenning, NSW, Australia); (2) ketoprofen 3 mg/kg IM (Troy Animal Health care, Troy laboratories, Glendenning, NSW, Australia); (3) ketoprofen 10 mg/kg topical (TD formulation from in vitro study). A one‐week ‘washout’ period was allowed between each treatment. Once all samples had been collected from the cattle administered TD ketoprofen (12 h), these animals were washed to remove any residual product using a commercially available shampoo (Mavlab Pty. Ltd, Logan City, QLD, Australia) applied with a soft brush, and thoroughly rinsed. The animals were not washed after receiving ketoprofen by either the IV or IM route. The cattle facilities used for handling of topically treated animals were appropriately washed down after all samples had been collected. Cattle receiving the IM and IV treatments were handled and housed using separate adjoining facilities away from any cattle treated with the topical treatment on the same day.

The TD formulation was prepared fresh on the morning of the study. Preliminary tests showed that this formulation was stable (20% ± 1%) at 0, 25 and 40°C for up to 6 months, although small amounts of ketoprofen methyl ester were detected in some samples stored at 20 and 40°C by 6 months (unpublished data). For application of the TD formulation, the test item was administered topically once to each animal as a single dose at the dose rate of 10 mg/kg (0.05 ml/kg). Each dose was individually calculated based on the animal's weight and rounded up to the most accurate graduation (0.2 ml) on the PP syringe. The test item was equilibrated to ambient temperature prior to administration. Cattle were dry at the time of treatment and remained dry until the completion of blood collection. The dose was administered as a pour‐on, using a suitably sized syringe (10 ml, Becton Dickinson, Australia), manually parting fur, and applied as a single band along the spine between the shoulders and approximately halfway along the back. Cattle were restrained in a standard cattle crush at the time of treatment. Personnel administering treatments wore personal protective equipment during administration, including elbow‐length gloves, fully enclosed boots and overalls. Gloves were replaced following each administration.

The animals were observed regularly during the bioavailability study. Following treatment on Day 0 and during the remainder of the working day, the animals were examined regularly for adverse reactions at the time of blood collections. Animals receiving the topical formulation were housed individually until the completion of blood collection on the day of administration and specifically observed for allogrooming behaviour. During the study, the cattle were inspected at least daily for general health and well‐being.

Blood samples (8 ml) were collected at regular intervals. For IV treatments, this was 0, 2, 5, 10, 15, 25 40 min, and then 1, 2, 3, 4, 6 and 8 h from the left side jugular using a 16 G 3″ catheter (the right jugular was used for IV administration, also via a 16 G 3″ catheter). For IM and TD administration, sample collection times were as follows: 0, 10, 20, 30 and 45 min, and then 1, 2, 4, 6, 8 and 12 h. Blood samples were collected into tubes containing lithium heparin and centrifuged, and the plasma was harvested and stored at −20°C for subsequent ketoprofen concentration analysis within 30 days.

#### 2.4.1 Pharmacokinetic analysis

Plasma ketoprofen concentrations were analysed for pharmacokinetic parameters, including maximum plasma concentration (*C*_MAX_), time to *C*_max_ (*T*_max_) following IM and TD Administrations, C_0_ for IV administration, area under the plasma–concentration–time curve from hour zero to the last quantifiable concentration (AUC_0‐Last_) or from time zero to infinity (AUC_inf_), terminal half‐life (T_½ᵦ_) using PKanalix (version 2021R1. Antony, France: Lixoft SAS) and non‐compartmental analysis. Bioavailability (F) was calculated by comparing the arithmetic mean of the dose‐normalized AUC_0‐Last_ following administration of the IM and TD formulation to the dose‐normalized AUC_0‐Last_ of the IV administration.

### 2.5 NSAID concentration analysis

Extraction: In vitro recipient or plasma samples were mixed with cold acetonitrile (0.2% formic acid) at 1:1 ratio and vortexed for 30 s to precipitate proteins. Centrifugation for 15 min at 20,000 × *g* was repeated twice with 30 min at 4°C between the centrifugation steps, to compact proteins into a pellet between each round. The supernatant was then collected, filtered through 0.22 μm nylon filter and analysed for ketoprofen concentration by tandem LCMS/MS. Deuterated ketoprofen (D3‐ketoprofen; CDN isotopes Inc. Pointe‐Claire, Quebec Canada) was used as an internal standard.

### 2.6 Analytical conditions

A Shimadzu Nexera uHPLC (Shimadzu Co. Ltd, Tokyo, Japan) consisting of two LC‐30 AD chromatographic pumps, CTO‐ 30A column oven, SIL‐30 AC autosampler and DGU‐20A5 degasser tandem with triple quadrupole Shimadzu LCMS 8030 was connected to medical grade argon gas (BOC Australia Ltd., Brisbane, QLD, Australia) into the collision cell and a nitrogen generator (NM32L, Peak Scientific Australia Pty Ltd, Melbourne, VIC, Australia) with an electrospray ion source operated on the positive ion mode in multiple reaction‐monitoring (MRM) detection mode.

The chromatographic separation was performed on a Phenomenex Kinetix Evo C18, 100 mm, 1.7 μm pore size column (Phenomenex Inc., Lane Cove, NSW, Australia) with a SecurityGuard™ Ultra Guard Cartridge (Phenomenex Inc., Lane Cove, NSW, Australia). A 5 μl sample injection volume was used with an elution conducted on a binary gradient from 15% Mobile Phase B held 0–0.2 min, then increased to 95% over 2.50 min then, 95% Mobile Phase B was held to 4.5 min elapse time before rapidly reducing Mobile Phase B back to 15% at 4.51 min and maintained at 15% until pump pressures were returned to stable initial column pressure. The total chromatographic separation was carried out over 7 min. Total flow rate of 400 μl/min between Pumps A and B was sustained with a column oven temperature retained at 40°C. Mobile phase A was 0.1% formic acid in water, and mobile phase B was 0.1% formic acid in acetonitrile. The mass spectrometer was operated using an electrospray ion source in the positive ion mode with the following instrument conditions: desolvation temperature at 250°C, heating block at 400°C, interface at 300°C with the nebulising gas flow at 2 L/min, with the drying gas flow of 10 L/min, and the capillary voltage set at 4.5 kv. The collision gas pressure was at 230 kPa, and collision energy was −12 V for both the analyte and internal standard. Dwell time for ion transitions was set between 80 and 100 milliseconds. Optimized multiple‐reaction‐monitoring quantifier/qualifier transitions for ketoprofen were m/z 255.10 > 105.20 and m/z 255.10 > 194.25. Transition ions for d3‐ketoprofen (ISTD) were m/z 258.10 > 212.10 and m/z 258.10 > 179.10. Pooled control blank bovine plasma matrix was spiked at 20 ng/ml and 100 ng/ml as analytical quality control samples prepared with experimental samples were inserted into the analytical batch every 20 samples. The linear calibration range used was between 2 ng/ml and 1 μg/ml.

### 2.7 Analytical method verification

Ketoprofen recovery after extractions was consistent at 91.2%–102.6% (mean ± SD). The limit of detection (LOD) was 0.3 ng/ml, and the linear calibration range was 0.019–5 μg/ml. The QC was determined over a range of concentrations (2, 20 and 100 μg/ml) and was between 9% to 11%.

### 2.8 Statistical analysis

Statistical analysis and graphing for the in vitro component of this study were conducted using Statistica™ version 13.5.0.17 (TIBCO Software Inc., Palo Alto, CA). In vitro excipient screening used Roy's largest root multivariate repeated measures ANOVA with across NSAID concentration, solvent/vehicle concentration against sampling time, where treatment, time and treatment–time interaction were entered as fixed effects. All reported *p* values are two‐tailed, with statistical significance defined as *p* < .05. In the post hoc tests, multiple analyses were corrected using the Bonferroni method. For the pharmacokinetic data, *C*_MAX_, *C*_MAX/dose_, *T*_max_ and AUC_0‐Last_ and AUC_inf/dose_ values for each cow were compared using R statistical programming. To compare the parameters between the TD and IM treatment groups, independent *t*‐tests were conducted, with assumptions of the independent *t*‐test, normality, and equal variance, were checked using, respectively, the Shapiro–Wilks test and Levene's test. However, for the comparison for AUC_inf/dose_, these assumptions were not met, so the Mann–Whitney *U*‐test was used instead.

## 3 RESULTS

Skin lesions were observed on all cattle at the site of TD application within 4–6 days of administration. Gross lesion observations included dermal thickening and flaking skin. Lesions were characterized by histopathology as a lichenoid lymphoplasmacytic dermatitis, with multifocal perivascular and periadnexal inflammation, basal cell vacuolation and apoptotic keratinocyte formation.

### 3.1 Formulation screening

The final TD formula consisted of 20% ketoprofen dissolved in a combination of 45%:45% ethanol and isopropyl myristate (IPM), mixed with 10% eucalyptus oil (~7% 1,8‐cineole). The full list of combinations tested can be found in A Appendix.

### 3.2 Bioavailability study

**FIGURE 1:**
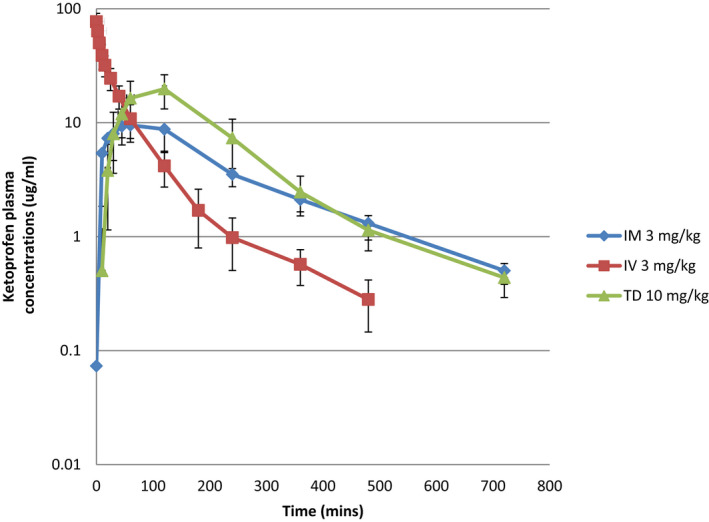
Plasma concentration‐time curves (mean ± SD) for ketoprofen administered to cattle by IV, IM and TD (topical) routes of administration

Since the vehicle (excipients) has a major role in determining the rate and extent of transdermal penetration drugs (Mills & Cross, 2006), the final formulation selected was based on overall penetration as measured by transdermal flux rates. Ketoprofen was first detected in the plasma approximately 10 min following administration of the topical formulation and the IM formulation (Figure 1, Table 1), but the corresponding plasma concentrations following TD administration were about 10‐fold lower than that following IM injection. However, the TD‐associated plasma concentrations rose quickly thereafter with an average *T*_MAX_ of ~2 h and more than twice the peak concentrations seen after IM injection. The *C*_MAX_ (*p* = .001), *T*_max_ (*p* = .01) and AUC_0‐Last_ (*p* = .002) were significantly higher after TD administration, compared to IM. However, if dose rate was factored in (3 mg/kg IM vs. 10 mg/kg TD), the *C*_MAX/dose_ (*p* = .001) and the AUC_inf/dose_ (*p* < .001) were significantly higher for IM compared to TD. AUC_inf_ percent extrapolation values were generally very low (2.1% ‐ 6.5%) with the exception of one animal that had greater than 20% (cow#160 for IM dosing), and as such, no AUCinf parameters were reported for this animal. The IM formulation had effectively complete bioavailability based on dose‐normalized AUC_inf_, while the TD bioavailability was 50%. The T½ᵦ for the IM and TD formulations were similar and were both slightly longer than the IV route. Individual plasma drug concentrations for each animal have been presented in C Appendix.

**TABLE 1:** Pharmacokinetic parameters for ketoprofen administered to cattle by IV, IM and TD routes

	Dose (mg/kg)		*C*_*MAX*_ *or C*_*0*_ (μg/ml)*	*C*_*MAX*_ /dose (μg/ml) / (mg/kg)	*T*_*MAX*_ (mins)	AUC_0‐Last_ (μg*min/ml)	AUC_inf_ (μg*min/ml)	AUC_inf_/dose (μg*min/ml)/(mg/kg)	T1/2β (mins)	F (%)
IV	3	Arithmetic mean	77	22	NA	2363	2418	806	121	
		Arithmetic SD	23	4.8	NA	569	591	197	64	
		Geometric mean	73	21	NA	2306	2357	786	110	
		Geometric SD	1.4	1.3	NA	1.3	1.3	1.3	1.5	
IM	3	Arithmetic mean	11	4	74	2376	2566	855	164	98
		Arithmetic SD	4.0	1.3	43	738	871	290	54	NA
		Geometric mean	10.8	3.6	59	2279	2445	815	157	
		Geometric SD	1.4	1.4	2.2	1.4	1.4	1.4	1.3	
Topical	10	Arithmetic mean	20	2	115	3940	4039	404	155	50
		Arithmetic SD	6.5	0.7	17	1324	1324	132	58	NA
		Geometric mean	19	2	113	3750	3856	386	148	
		Geometric SD	1.4	1.4	1.2	1.4	1.4	1.4	1.4	

a *C*_*MAX*_ for TD and IM dosing. C0 reported for IV dosing, calculated from back extrapolation to time 0.

## 4 DISCUSSION

A major outcome from the current study was that ketoprofen could be applied topically to cattle and achieve a similar plasma drug profile as IM administration, albeit at 3x the IM dose. The TD formulation was easy to administer, with a low volume (~ 10 ml) applied to the backline making it highly convenient to apply to cattle in a race or crush. Avoiding a parenteral route of administration removed concerns relating to broken needles and reduced meat quality following bruising and/or introduction of microbes from needles. The surprisingly short lag time (appearance of ketoprofen concentrations in plasma) rivalled IM administration, and plasma concentrations of both groups were similar at 30 min suggesting that comparable efficacy to the IM formulation is possible when using this TD formulation and dose. However, efficacy studies would be necessary to verify this.

It has been shown than pre‐emptive analgesia is far more effective and likely to avoid negative pathophysiological changes, compared to analgesia applied after tissue injury (Kaka et al., 2018). NSAIDs can significantly reduce the pain response following surgery by limiting the inflammatory changes induced by surgery (Stafford & Mellor, 2005). In pigs, topical anaesthesia provided only minor benefit compared to parenteral NSAIDs injected prior to castration. However, markers of pain were most prominent within the first hour after surgery, so any analgesic must act quickly after administration to provide maximum benefit (Gottardo et al., 2016). Another significant outcome from the current study was the relatively high bioavailability (~50%) of active drug from TD administration. This is substantially higher than has been reported for NSAIDs in humans (Herkenne et al., 2008; Kienzler et al., 2010). Indeed, many topical NSAID formulations intended for human use target local drug concentrations under the site of application, such as a knee joint, and base efficacy on clinical improvement (Kienzler et al., 2010).

The findings in the current study support what has been shown for human medicine, with ketoprofen (and diclofenac, although the latter is not registered for use in cattle) having the highest penetration through human skin (Cordero et al., 1997; Derry et al., 2017; Mazieres, 2005). TD ketoprofen has proven useful to treat acute pain resulting for sprains or strains in humans (Derry et al., 2017), while a ketoprofen gel was superior to placebo at 30 min after being applied to sprained ankles (Serinken et al., 2016). Studies in rats have similarly demonstrated that ketoprofen is superior to other NSAIDs for providing pain relief from TD application because of its higher skin permeability (Amagai et al., 2013).

In veterinary species, TD flunixin meglumine has proven successful to successfully penetrate through the skin of cattle and achieve systemic therapeutic concentrations (Kleinhenz et al., 2016, Kleinhenz, Gorden, et al., 2018, Kleinhenz, Van Engen, et al., 2018). However, TD flunixin meglumine was less successful in penetrating through the skin of meat goats (Reppert et al., 2019a), alpacas (Reppert et al., 2019b) and mature sows (Cramer et al., 2019).

One aspect of the current study that was concerning was the appearance of skin irritation under the site of application. This appeared to be self‐limiting and improved over time. It was initially thought that this may be related to the potential of ketoprofen to photosensitize skin (Bagheri et al., 2000). Similarly, local skin reactions have been reported in humans following topical NSAID administration (Bonifacio et al., 2018), particularly when diclofenac was combined with a menthol vehicle (Moreira & Liu, 2017), although these adverse events were generally mild and transient. The cause of the skin lesions observed in all cattle following application of the TD formulation is uncertain at this time. A photosensitive dermatitis associated with a photoallergic reaction to ketoprofen has been described in other species (Nakazawa et al., 2006). However, the impact of washing with a detergent at the treatment sites could be a contributing factor. We note that skin irritation was not seen during pilot studies (unpublished results). Further investigations to establish repeatability and causality of the skin lesions with the transdermal ketoprofen formulation in cattle are indicated.

The analgesic activity of NSAIDs, including ketoprofen, is affected by their ability to limit the inflammatory changes induced by tissue damage (Stafford & Mellor, 2005; Viñuela‐Fernández et al., 2007). Several studies have combined ketoprofen (3 mg/kg IV) with local anaesthetics (injected prior to the procedure) and this virtually eliminated the cortisol responses to pain caused by soft tissue surgery, such as castration (Stafford et al., 2002; Stafford & Mellor, 2005). Furthermore, systemic ketoprofen alone provided effective analgesia in bull calves following castration (Earley & Crowe, 2002; Stafford et al., 2002b; Ting et al., 2003a, 2003b). The efficacy of ketoprofen analgesia during castration may relate to its acting on other sites, including centrally, that are not affected by local anaesthetic, plus it has a strong anti‐inflammatory effect post‐operatively (Stafford & Mellor, 2005). Similarly, the cortisol response was delayed until 5 h after dehorning when ketoprofen but not when phenylbutazone was administered, which may relate to ketoprofen‐sensitive and cortisol‐sensitive sensory input (Sutherland et al., 2002). Irrespective, while ketoprofen may not prevent the initial pain response during the first 1 h following dehorning, it does appear useful in ameliorating the subsequent inflammation‐induced pain response (McMeekan et al., 1998). Importantly, it is well recognized that aggressive management of acute pain is preferable and reduces the likelihood of the development of maladaptive (neuropathic) pain which is difficult to control with conventional analgesics (Viñuela‐Fernández et al., 2007). As such, and consistent with early studies on surgical husbandry procedures in cattle, a combination of a NSAID with local or regional analgesia provides the most effective control of pain (McMeekan et al., 1998; Stafford et al., 2002; Sutherland et al., 2002).

A further advantage of TD ketoprofen is the better risk‐to‐benefit ratio than NSAIDs administered by other routes, including oral (Rafanan et al., 2018). A reduction in gastric irritation and first pass metabolism, minimal invasiveness and enhanced owner compliance are all hallmarks of the advantaged of topical administration over other routes (Mills & Cross, 2006). Ketoprofen delivered by a transdermal patch during Phase III clinical trials in human patients with non‐articular rheumatism and traumatic painful soft tissue injuries was significantly more effective than placebo at reducing pain during daily activities. This was attributed to tissue drug levels remaining high, while systemic drug concentrations were sufficiently low to reduce the risk of systemic adverse events caused by elevated serum NSAID levels (Mazieres, 2005).

In summary, results indicate that this novel TD formulation provides a substantial dosing convenience which may improve animal welfare and achieves similar plasma pharmacokinetics to the IM product at when given at doses of 10 mg/kg. Further research in cattle undergoing painful procedures related to routine husbandry practices is required to evaluate the analgesic effect of the TD formulation in comparison with the established efficacy of the IM product. Once validated, the TD formulation could be a significant therapeutic tool for addressing the unmet need of analgesia during the post‐procedural period with an efficacious, needle‐free product.

## AUTHOR CONTRIBUTIONS

All authors have read and approved the final manuscript. PCM, JGO, JBR, MM and NS contributed to conception, experimental design analysis and drafted the manuscript. CB ran the study itself and performed all sampling. SW provided statistical analysis. All authors critically revised the manuscript, provided final approval, and agreed to be accountable for all aspects of work ensuring integrity and accuracy.

## FUNDING INFORMATION

This project was made possible by funding from Elanco Australasia Pty Ltd and Meat and Livestock Australia (MLA).

## CONFLICT OF INTEREST

Some of the authors work at Elanco Animal Health, Greenfield, IN, USA (Owens, Reinbold) or Elanco Australasia (Bergner).

## ANIMAL WELFARE AND ETHICS STATEMENT

The in vitro screening study was approved by The University of Queensland Animal Ethics Committee (approval certificate SVS/455/14/MLA). The in vivo bioavailability study was approved by the Elanco Animal Ethics Committee and conducted under the APVMA Small‐scale Trials Permit PER7250 and GLP study conditions. The authors confirm that the ethical policies of the journal, as noted on the journal's author guidelines page, have been adhered to and that the appropriate ethical review committee approval has been received. The authors confirm that they have adhered to International Standards for the protection of animals used for scientific purposes.

## Data Availability

The data that support the findings of this study are available in the supplementary material of this article.

## APPENDIX—List of vehicles (excipients) screened for ketoprofen A

**Table:**

	Ketoprofen (%_(w/v)_)	Solvent base	Vehicle	Terpene
1	10	50% MeOH	none	none
2	20	50% MeOH	none	none
3	50	50% MeOH	none	none
4	10	50% EthOH	none	none
5	20	50% EthOH	none	none
6	50	50% EthOH	none	none
7	10	50% IPN	none	none
8	20	50% IPN	none	none
9	50	50% IPN	none	none
10	10	30% EthOH:MeOH	none	none
11	20	30% EthOH:MeOH	none	none
12	50	30% EthOH:MeOH	none	none
13	10	50% EthOH:IPN	none	none
14	20	50% EthOH:IPN	none	none
15	50	50% EthOH:IPN	none	none
16	10	70% EthOH:IPN	none	none
17	20	70% EthOH:IPN	none	none
18	50	70% EthOH:IPN	none	none
19	10	45% EthOH	1‐ethyl‐2‐Pyrrolidinone	10% 1,8‐cineole
20	10	45% EthOH	Isopropyl myristate	10% 1,8‐cineole
21	10	45% EthOH	Propylene glycol	10% 1,8‐cineole
22	10	50% EthOH	none	10% 1,8‐cineole
23	10	50% EthOH	Isopropyl myristate	5% Eucalyptus oil
24	10	50% EthOH	Isopropyl myristate	5% 1,8‐cineole
25	10	50% EthOH	Isopropyl myristate	5 mg/ml Menthol
26	10	50% EthOH	1‐Ethyl‐2‐Pryrrolidone	5% Eucalyptus oil
27	10	50% EthOH		5 mg/ml Menthol
28	10	50% EthOH	1‐ethyl‐2‐Pyrrolidinone	5% 1,8‐cineole
29	10	50% EthOH		5% Eucalyptus oil
30	10	50% EthOH	Oleyl alcohol	5% 1,8‐cineole
31	10	50% EthOH		5% Eucalyptus oil
32	10	50% EthOH		5 mg/ml Menthol
33	10	50% EthOH	Propylene glycol	5% 1,8‐cineole
34	10	50% EthOH		5% Eucalyptus oil
35	10	50% EthOH		5 mg/ml Menthol
36	10	50% IPN	none	None
37	10	50% IPN	Isopropyl myristate	5% Eucalyptus oil
38	10	50% IPN	Isopropyl myristate	5 mg/ml Menthol
39	10	50% IPN	Isopropyl myristate	5% 1,8‐cineole
40	10	50% IPN	Isopropyl myristate	10% 1,8‐cineole
41	10	50% IPN	1‐Ethyl‐2‐Pryrrolidone	5% Eucalyptus oil
42	10	50% IPN		5 mg/ml Menthol
43	10	50% IPN	1‐ethyl‐2‐Pyrrolidinone	5% 1,8‐cineole
44	10	50% IPN		5% Eucalyptus oil
45	10	50% IPN	Oleyl alcohol	5% 1,8‐cineole
46	10	50% IPN		5% Eucalyptus oil
47	10	50% IPN		5 mg/ml Menthol
48	10	50% IPN	Propylene glycol	5% 1,8‐cineole
49	10	50% IPN		5% Eucalyptus oil
50	10	50% IPN		5 mg/ml Menthol
51	10	70% EthOH	Oleyl alcohol	None
52	10	70% EthOH	Propylene glycol	None
53	10	70% EthOH	Propylene glycol	5 mg/ml Menthol
54	10	70% IPN	none	None
55	10	30% EthOH	70% Isopropyl myristate	5% Eucalyptus oil
56	10	30% EthOH	70% Isopropyl myristate	10% Eucalyptus oil
57	10	50% EthOH	50% Isopropyl myristate	5% Eucalyptus oil
58	10	50% EthOH	50% Isopropyl myristate	10% Eucalyptus oil
59	10	70% EthOH	30% Isopropyl myristate	5% Eucalyptus oil
60	10	70% EthOH	30% Isopropyl myristate	10% Eucalyptus oil
61	10	None	none	100% Eucalyptus oil
62	10	None	Crodamol STS‐LQ(MH)	None
63	10	None	Crodamol‐STS‐LQ	None
64	10	None	Cromollient‐SCE‐LQ‐(MH)	None
65	10	None	Propylene glycol	None
66	10	None	Superfine Arlasolve	None
67	10	None	70% Propylene glycol	5% Eucalyptus oil
68	10	None	70% Propylene glycol	10% Eucalyptus oil
69	10	None	70% Propylene glycol	5% Eucalyptus oil
70	10	None	50% Propylene glycol	10% Eucalyptus oil
71	10	None	50% Propylene glycol	5% Eucalyptus oil
72	10	None	30% Propylene glycol	10% Eucalyptus oil
73	10	20% EthOH	70% Isopropyl myristate	5% Eucalyptus oil
74	10	20% EthOH	70% Isopropyl myristate	10% Eucalyptus oil
75	10	20% EthOH	50% Isopropyl myristate	5% Eucalyptus oil
76	10	20% EthOH	50% Isopropyl myristate	10% Eucalyptus oil
77	10	20% EthOH	30% Isopropyl myristate	5% Eucalyptus oil
78	10	20% EthOH	30% Isopropyl myristate	10% Eucalyptus oil
79	10	20% EthOH	70% Propylene glycol	5% Eucalyptus oil
80	10	20% EthOH	70% Propylene glycol	10% Eucalyptus oil
81	10	20% EthOH	50% Propylene glycol	5% Eucalyptus oil
82	10	20% EthOH	50% Propylene glycol	10% Eucalyptus oil
83	10	20% EthOH	30% Propylene glycol	5% Eucalyptus oil
84	10	20% EthOH	30% Propylene glycol	10% Eucalyptus oil
85	10	45% EthOH	70% Isopropyl myristate	5% Eucalyptus oil
86	10	45% EthOH	70% Isopropyl myristate	10% Eucalyptus oil
87	10	45% EthOH	50% Isopropyl myristate	5% Eucalyptus oil
88	10	45% EthOH	50% Isopropyl myristate	10% Eucalyptus oil
89	10	45% EthOH	30% Isopropyl myristate	5% Eucalyptus oil
90	10	45% EthOH	30% Isopropyl myristate	10% Eucalyptus oil
91	10	45% EthOH	70% Propylene glycol	5% Eucalyptus oil
92	10	45% EthOH	70% Propylene glycol	10% Eucalyptus oil
93	10	45% EthOH	50% Propylene glycol	5% Eucalyptus oil
94	10	45% EthOH	50% Propylene glycol	10% Eucalyptus oil
95	10	45% EthOH	30% Propylene glycol	5% Eucalyptus oil
96	10	45% EthOH	30% Propylene glycol	10% Eucalyptus oil
97	10	50% EthOH	none	5 mg/ml Menthol
98	10	50% EthOH	50% Isopropyl myristate	5 mg/ml Menthol
99	10	50% EthOH	50% Propylene glycol	5 mg/ml Menthol
100	10	50% IPN	none	5 mg/ml Menthol
101	10	50% IPN	50% Isopropyl myristate	5 mg/ml Menthol
102	10	50% IPN	50% Propylene glycol	5 mg/ml Menthol
103	10	70% EthOH	none	5 mg/ml Menthol
104	10	70% EthOH	50% Isopropyl myristate	5 mg/ml Menthol
105	10	70% EthOH	50% Propylene glycol	5 mg/ml Menthol
106	10	70% IPN	none	5 mg/ml Menthol
107	10	70% IPN	50% Isopropyl myristate	5 mg/ml Menthol
108	10	70% IPN	50% Propylene glycol	5 mg/ml Menthol

## APPENDIX—Individual plasma concentration and pharmacokinetic data for each cow following IV, IM or TD administration of ketoprofen to cattle (mean ± SD) C

**Table:**

	Time	# 10	# 183	# 184	# 176	# 128	# 169	# 14	# 11	# 181	# 178	# 175	# 160	Mean	SD
IM	0	0.00	0.00	0.00	0.00	0.00	0.00	0.00	0.00	0.00	0.00	0.00	0.88	0.07	0.25
	10	2.42	3.89	5.69	1.70	7.44	2.80	7.44	3.88	8.01	3.70	13.96	3.84	5.40	3.41
	20	4.12	4.80	11.22	2.15	11.48	6.62	12.42	5.93	8.36	4.50	11.24	4.75	7.30	3.51
	30	3.35	6.56	13.09	3.41	12.87	7.47	12.86	5.99	7.19	6.20	11.01	6.18	8.02	3.55
	45	5.46	7.59	13.00	8.07	19.94	7.09	13.67	6.24	6.81	7.34	10.51	6.19	9.33	4.27
	60	7.96	7.52	13.23	4.13	16.77	8.18	16.68	7.83	6.46	10.07	10.04	5.20	9.51	4.11
	120	9.80	8.87	12.97	4.89	9.93	9.16	12.18	10.84	4.29	11.81	6.69	3.78	8.77	3.16
	240	3.25	2.90	1.91	3.49	4.14	2.39	5.41	3.10	2.36	4.81	4.74	3.72	3.52	1.09
	360	1.98	1.27	1.96	2.18	3.32	1.00	2.09	1.73	1.67	1.76	3.85	2.56	2.11	0.80
	480	0.90	0.67	0.65	0.87	3.39	1.08	1.15	0.75	0.72	0.93	2.91	1.70	1.31	0.91
	720	0.37	0.27	0.19	0.24	1.30	0.20	0.99	0.25	0.28	0.23	1.70	0.00	0.50	0.53
IV	0	0.00	0.00	0.00	0.00	0.00	0.00	0.00	0.07	0.00	0.00	0.00	0.00	0.01	0.02
	2	58.77	47.90	64.19	43.82	78.64	53.48	81.67	83.00	62.28	84.03	67.55	39.83	63.76	15.64
	5	40.65	50.74	49.88	36.37	54.81	38.38	75.36	63.87	35.82	51.12	57.08	47.81	50.16	11.77
	10	37.55	34.16	39.52	NR	46.36	30.21	52.84	56.66	35.39	38.42	24.28	32.58	38.90	9.65
	15	29.07	30.15	40.08	29.21	26.29	23.71	35.25	44.41	33.41	35.57	21.32	34.32	31.90	6.64
	25	20.47	19.99	23.60	19.40	25.29	23.67	27.72	37.71	26.92	28.86	18.60	21.74	24.50	5.37
	40	15.23	13.29	16.50	15.88	16.90	12.37	22.42	25.83	17.71	19.82	15.48	13.54	17.08	3.93
	60	8.98	8.32	13.03	6.02	10.89	12.18	13.85	17.18	10.99	14.35	9.34	4.82	10.83	3.57
	120	3.72	2.18	4.25	3.41	3.53	4.99	5.20	7.03	6.03	4.16	3.35	2.21	4.17	1.45
	180	1.97	0.89	1.71	1.13	1.50	2.16	1.41	4.26	1.75	1.70	1.06	0.88	1.70	0.91
	240	1.20	0.60	0.78	0.71	0.87	1.02	0.94	2.33	0.95	1.17	0.75	0.46	0.98	0.48
	360	0.50	0.32	0.58	0.61	0.38	0.92	0.56	0.84	0.75	0.63	0.28	0.48	0.57	0.20
	480	0.19	0.19	0.44	0.15	0.26	0.47	0.48	0.43	0.23	0.26	0.12	0.16	0.28	0.14
Topical	0	0.00	0.00	0.00	0.00	0.00	0.00	0.00	0.00	0.00	0.00	0.00	0.00	0.00	0.00
	10	0.42	0.29	0.03	0.38	0.12	0.12	0.35	0.02	0.27	1.92	1.89	0.21	0.50	0.67
	20	4.61	3.77	0.84	2.80	2.93	2.31	2.79	2.21	1.92	9.71	8.50	3.27	3.80	2.66
	30	7.40	7.45	2.68	4.63	7.27	7.47	6.75	5.78	5.94	19.64	12.83	7.86	7.97	4.38
	45	7.81	10.81	6.36	9.94	12.40	10.49	14.10	10.96	9.49	23.30	17.18	10.09	11.91	4.55
	60	10.54	14.81	9.57	9.65	16.45	16.57	18.50	17.02	13.83	26.12	31.87	12.01	16.41	6.70
	120	10.09	18.88	14.03	17.45	27.06	18.03	18.98	18.49	17.26	26.34	34.57	15.99	19.76	6.56
	240	2.46	7.16	5.28	7.73	14.66	5.56	6.65	4.47	6.11	6.33	12.49	9.16	7.34	3.39
	360	0.79	2.85	2.12	2.10	4.50	2.19	2.28	2.05	1.69	2.46	3.62	2.85	2.46	0.94
	480	0.59	1.58	1.10	0.80	1.81	1.33	0.85	0.64	1.10	1.37	1.55	0.96	1.14	0.39
	720	0.36	0.71	0.41	0.27	0.67	0.50	0.35	0.31	0.30	0.57	0.40	0.40	0.44	0.14

NR: not reported.

**Table:**

Animal#	Route	C_0_	C_MAX_	C_MAX_ /dose	T_MAX_	AUC_0‐Last_	AUC_inf_	AUC%extrapolated	AUC_inf_/dose	T1/2β
		(μg/ml)	(μg/ml)	(μg/ml) / (mg/kg)	(mins)	(μg*min/ml)	(μg*min/ml)	(%)	(μg*min/ml) / (mg/kg)	(mins)
# 10	IV	75.14	58.77	19.59	NA	2140.27	2164.85	1.14	721.62	89.68
# 10	IM	NA	9.8	3.27	120	2108.68	2188.97	3.67	729.66	150.42
# 10	TD	NA	10.54	1.05	60	1976.91	2143.16	7.76	214.32	320.11
# 11	IV	98.84	83	27.67	NA	3711.68	3766.04	1.44	1255.35	87.63
# 11	IM	NA	10.84	3.61	120	2159.13	2206.16	2.13	735.39	130.38
# 11	TD	NA	18.49	1.85	120	3263.95	3319.06	1.66	331.91	123.24
# 128	IV	100.04	78.64	26.21	NA	2361.32	2405.84	1.85	801.95	118.69
# 128	IM	NA	19.94	6.65	45	3723.33	4095.58	9.09	1365.19	198.48
# 128	TD	NA	27.06	2.71	120	5825.76	5956.46	2.19	595.65	135.22
# 14	IV	86.17	81.67	27.22	NA	2905.4	3040.3	4.44	1013.43	194.8
# 14	IM	NA	16.68	5.56	60	3413.32	3644.99	6.36	1215	162.21
# 14	TD	NA	18.98	1.9	120	3800.27	3858.27	1.5	385.83	114.86
# 160	IV	39.83	47.81	15.94	NA	1698.75	1720.54	1.27	573.51	94.43
# 160	IM	NA	6.19	2.06	45	1640.88	NR	NR	NR	212.43
# 160	TD	NA	15.99	1.6	120	3696.12	3758.49	1.66	375.85	108.09
# 169	IV	66.72	53.48	17.83	NA	2278.73	2330.65	2.23	776.88	76.57
# 169	IM	NA	9.16	3.05	120	1921.59	1962.55	2.09	654.18	141.96
# 169	TD	NA	18.03	1.8	120	3553.86	3675.84	3.32	367.58	169.1
# 175	IV	75.58	67.55	22.52	NA	1935.99	1952.19	0.83	650.73	93.55
# 175	IM	NA	13.96	4.65	10	3253.01	4003.15	18.74	1334.38	305.86
# 175	TD	NA	34.57	3.46	120	6712.87	6778.97	0.98	677.9	114.55
# 176	IV	49.62	43.82	14.61	NA	1818.59	1837.09	1.01	612.36	85.5
# 176	IM	NA	8.07	2.69	45	1621.16	1663.03	2.52	554.34	120.94
# 176	TD	NA	17.45	1.75	120	3353.84	3402.66	1.43	340.27	125.33
# 178	IV	117.04	84.03	28.01	NA	2724.4	2766.68	1.53	922.23	112.74
# 178	IM	NA	11.81	3.94	120	2577.11	2617.62	1.55	872.54	122.1
# 178	TD	NA	26.34	2.63	120	5098.88	5241.25	2.72	524.13	173.13
# 181	IV	90.05	62.28	20.76	NA	2492.46	2513.4	0.83	837.8	63.11
# 181	IM	NA	8.36	2.79	20	1596.31	1655.05	3.55	551.68	145.42
# 181	TD	NA	17.26	1.73	120	3288.71	3350.07	1.83	335.01	141.77
# 183	IV	47.9	50.74	16.91	NA	1808.47	1848.13	2.15	616.04	144.67
# 183	IM	NA	8.87	2.96	120	1926.76	1990.63	3.21	663.54	163.96
# 183	TD	NA	18.88	1.89	120	3948.98	4136.55	4.53	413.66	183.12
# 184	IV	75.94	64.19	21.4	NA	2484.7	2669.15	6.91	889.72	290.57
# 184	IM	NA	13.23	4.41	60	2570.17	2600.38	1.16	866.79	110.22
# 184	TD	NA	14.03	1.4	120	2761.7	2852.83	3.19	285.28	154.05

NR: not reported.
